# Using Text Content From Coronary Catheterization Reports to Predict 5-Year Mortality Among Patients Undergoing Coronary Angiography: A Deep Learning Approach

**DOI:** 10.3389/fcvm.2022.800864

**Published:** 2022-02-28

**Authors:** Yu-Hsuan Li, I-Te Lee, Yu-Wei Chen, Yow-Kuan Lin, Yu-Hsin Liu, Fei-Pei Lai

**Affiliations:** ^1^Department of Computer Science & Information Engineering, National Taiwan University, Taipei, Taiwan; ^2^Division of Endocrinology and Metabolism, Department of Internal Medicine, Taichung Veterans General Hospital, Taichung, Taiwan; ^3^School of Medicine, National Yang-Ming University, Taipei, Taiwan; ^4^School of Medicine, Chung Shan Medical University, Taichung, Taiwan; ^5^Cardiovascular Center, Taichung Veterans General Hospital, Taichung, Taiwan; ^6^Department of Computer Science, Columbia University, New York, NY, United States; ^7^Graduate Institute of Biomedical Electronics and Bioinformatics, National Taiwan University, Taipei, Taiwan; ^8^Department of Electrical Engineering, National Taiwan University, Taipei, Taiwan

**Keywords:** coronary catheterization reports, coronary angiography, mortality, deep learning, natural language processing

## Abstract

**Background:**

Current predictive models for patients undergoing coronary angiography have complex parameters which limit their clinical application. Coronary catheterization reports that describe coronary lesions and the corresponding interventions provide information of the severity of the coronary artery disease and the completeness of the revascularization. This information is relevant for predicting patient prognosis. However, no predictive model has been constructed using the text content from coronary catheterization reports before.

**Objective:**

To develop a deep learning model using text content from coronary catheterization reports to predict 5-year all-cause mortality and 5-year cardiovascular mortality for patients undergoing coronary angiography and to compare the performance of the model to the established clinical scores.

**Method:**

This retrospective cohort study was conducted between January 1, 2006, and December 31, 2015. Patients admitted for coronary angiography were enrolled and followed up until August 2019. The main outcomes were 5-year all-cause mortality and 5-year cardiovascular mortality. In total, 11,576 coronary catheterization reports were collected. BioBERT (bidirectional encoder representations from transformers for biomedical text mining), which is a BERT-based model in the biomedical domain, was utilized to construct the model. The area under the receiver operating characteristic curve (AUC) was used to assess model performance. We also compared our results to the residual SYNTAX (SYNergy between PCI with TAXUS and Cardiac Surgery) score.

**Results:**

The dataset was divided into the training (60%), validation (20%), and test (20%) sets. The mean age of the patients in each dataset was 65.5 ± 12.1, 65.4 ± 11.2, and 65.6 ± 11.2 years, respectively. A total of 1,411 (12.2%) patients died, and 664 (5.8%) patients died of cardiovascular causes within 5 years after coronary angiography. The best of our models had an AUC of 0.822 (95% CI, 0.790–0.855) for 5-year all-cause mortality, and an AUC of 0.858 (95% CI, 0.816–0.900) for 5-year cardiovascular mortality. We randomly selected 300 patients who underwent percutaneous coronary intervention (PCI), and our model outperformed the residual SYNTAX score in predicting 5-year all-cause mortality (AUC, 0.867 [95% CI, 0.813–0.921] vs. 0.590 [95% CI, 0.503–0.684]) and 5-year cardiovascular mortality (AUC, 0.880 [95% CI, 0.873–0.925] vs. 0.649 [95% CI, 0.535–0.764]), respectively, after PCI among these patients.

**Conclusions:**

We developed a predictive model using text content from coronary catheterization reports to predict the 5-year mortality in patients undergoing coronary angiography. Since interventional cardiologists routinely write reports after procedures, our model can be easily implemented into the clinical setting.

## Introduction

For patients undergoing coronary angiography, their prognosis varies after the procedure. During coronary angiography, patients with obstructive coronary artery disease (CAD) would receive percutaneous coronary intervention (PCI), but the mortality rate is reportedly as high as 10–40% after the procedure (1). For patients with non-obstructive CAD noted during coronary angiography, their cardiovascular mortality is still higher than that of the general population (2). Current guidelines have suggested several predictive tools for patients with varied degrees of stenosis found during coronary angiography (3). The SYNergy between PCI with TAXUS and Cardiac Surgery (SYNTAX) score (4), and residual SYNTAX score (RSS) (5) are both used to predict adverse outcomes in patients with complex CAD. The Global Registry of Acute Coronary Events (GRACE) score was developed to predict in-hospital mortality in patients with acute coronary syndrome (ACS) (6). Recently, a surge in machine learning models has emerged for patients with both obstructive and non-obstructive CAD to predict their outcomes in various clinical settings (7). However, with all these predictive tools, clinicians are overwhelmed due to the need for familiarity with the models to choose the correct predictive tool for their patients. Furthermore, current models require human experts to extract the parameters from electronic health records (EHRs) to calculate the score, which is time consuming and limits their clinical application.

On the other hand, coronary angiography reports provide abundant information regarding the patients who have undergone the procedure. According to the statement regarding coronary catheterization reports published in 2014 (8), coronary angiography reports should include the indication for the procedure, a brief personal history of CAD, hemodynamic data during the procedure, descriptions of the coronary angiographic lesions, technique for revascularization, conclusions, and recommendations after PCI. Owing to recent advances in deep learning and natural language processing (NLP) (9), we can use free-text reports as inputs without choosing parameters before constructing a model. One of the state-of-the-art deep learning NLP algorithms is the bidirectional encoder representations from transformers (BERT) (9). The BERT model is pre-trained with a large text corpus and can be fine-tuned for a wide range of tasks, such as classification, question answering, and natural language understanding. As for the biomedical field, BioBERT (10) was developed and was trained with PubMed Central free text and PubMed abstract to comprehend biomedical texts. In this study, we used the BioBERT model with the text content of coronary catheterization reports to predict the 5-year all-cause mortality and the 5-year cardiovascular mortality among patients undergoing coronary angiography.

## Materials and Methods

### Datasets

Data were collected from patients who were admitted to Taichung Veterans General Hospital between January 2006 and December 2015 for coronary angiography. Patients were excluded if they were admitted for peripheral vascular catheterization, cerebrovascular catheterization, valvular heart disease, congenital structural heart disease, or arrhythmia. The contents of the reports are in compliance with the statement published by the American College of Cardiology Foundation, the American Heart Association, and the Society for Cardiovascular Angiography, and Interventions Foundation (8) which suggest reports should include the following information: a description of the procedure's indication; a brief history of the patient, hemodynamic data, coronary artery lesions, and the percentage of stenosis; thrombosis and myocardial infarction flow; treatment target lesions; equipment used; results of the intervention; and a conclusion to summarize the intervention and future recommendations for the patient.

Patient demographics such as age, sex, and date of the procedure were also collected. The mortality data (up to August 31, 2019) were retrieved from the Collaboration Center of Health Information Application, Department of Health, Executive Yuan, Taiwan, served as the primary outcome. The study complied with principles of the Declaration of Helsinki and was approved by the Institutional Review Board of Taichung Veterans General Hospital.

### Model Development and Evaluation

**Figure 1:**
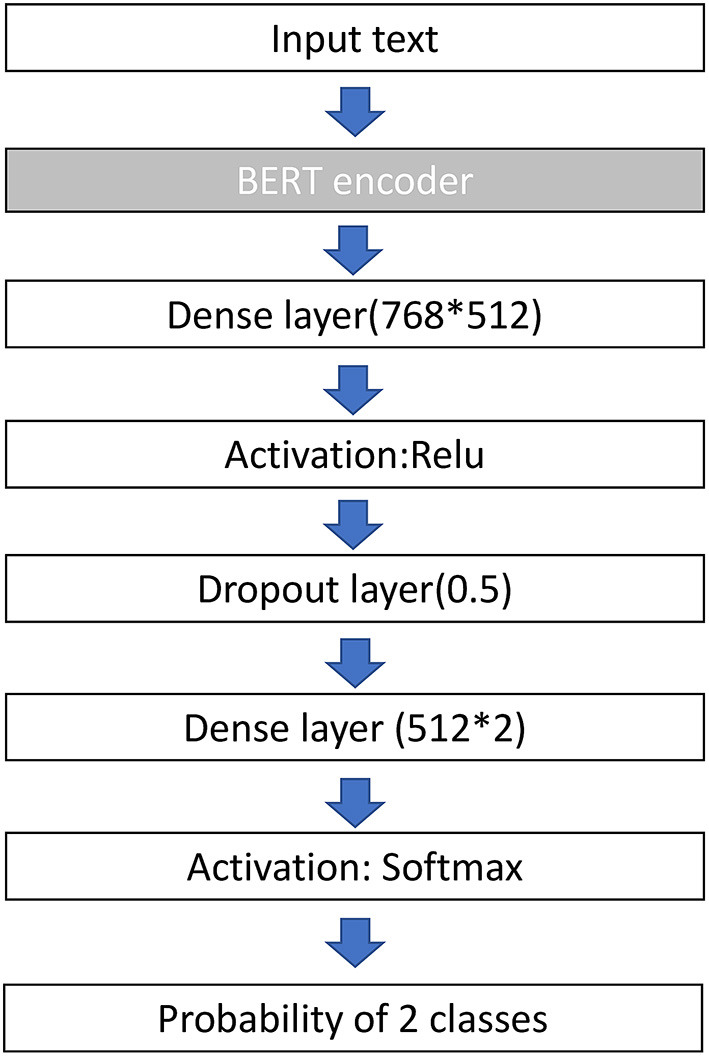
Architecture of the BERT model used in this study. BERT, bidirectional encoder representations from transformers; ReLu, rectified linear unit.

Before the development of our model, all reports were preprocessed by the Natural Language Tool Kit (NLTK) library to remove punctuation, change text to lower case, and remove stop words. The NLTK library is an open-source project that has abundant resources for language preprocessing. We then randomly divided the reports into training data (60%), validation data (20%), and testing data (20%). BioBERT was utilized as the baseline deep learning NLP architecture in our study. Inherited from Transformer's architecture (11), BioBERT has a multilayer bidirectional transformer encoder that includes 12 layers (transformer blocks), 768 hidden size, and 12 self-attention heads. It was pre-trained with PubMed abstracts and PubMed Central full-text articles to contextualize biomedical texts and can be fine-tuned for classification, question answering, and translation. We added one dense layer sized 768 × 512, followed by a dropout layer of 0.5, and a dense layer of 512 × 2 to fine-tune our text reports. The AdamW optimizer served as the learning rate adaptor, with an initial learning rate of 2e-5 and a batch size of 32. We used cross-entropy loss as the loss function. We trained the model for a maximum of seven epochs and selected the model with the minimum validation loss. The validation and training losses both decreased gradually during training of the epochs, which indicated no signs of overfitting. Due to the imbalance classes in our dataset (alive and dead ratio 7:1), we reweighed the classes accordingly (alive and dead ratio 1:7) to improve performance. The architecture of the proposed model is illustrated in Figure 1.

Because the maximum input for the BERT-base model is 512 tokens, we divided the text reports into three parts, including indication (indication of coronary angiography and brief history of the patients), technique (hemodynamic data, coronary artery lesion and severity, equipment, and intervention), and conclusion (summary of this procedure and recommendation for follow-up). Next, we fine-tuned our baseline model with each part of the text reports separately. To improve model performance, we used a linear combination of the probability from each model to produce the result, referred to in this study as the ensemble model. The performance was evaluated using the area under the receiver operating characteristic curve (AUC).

**Figure 2:**
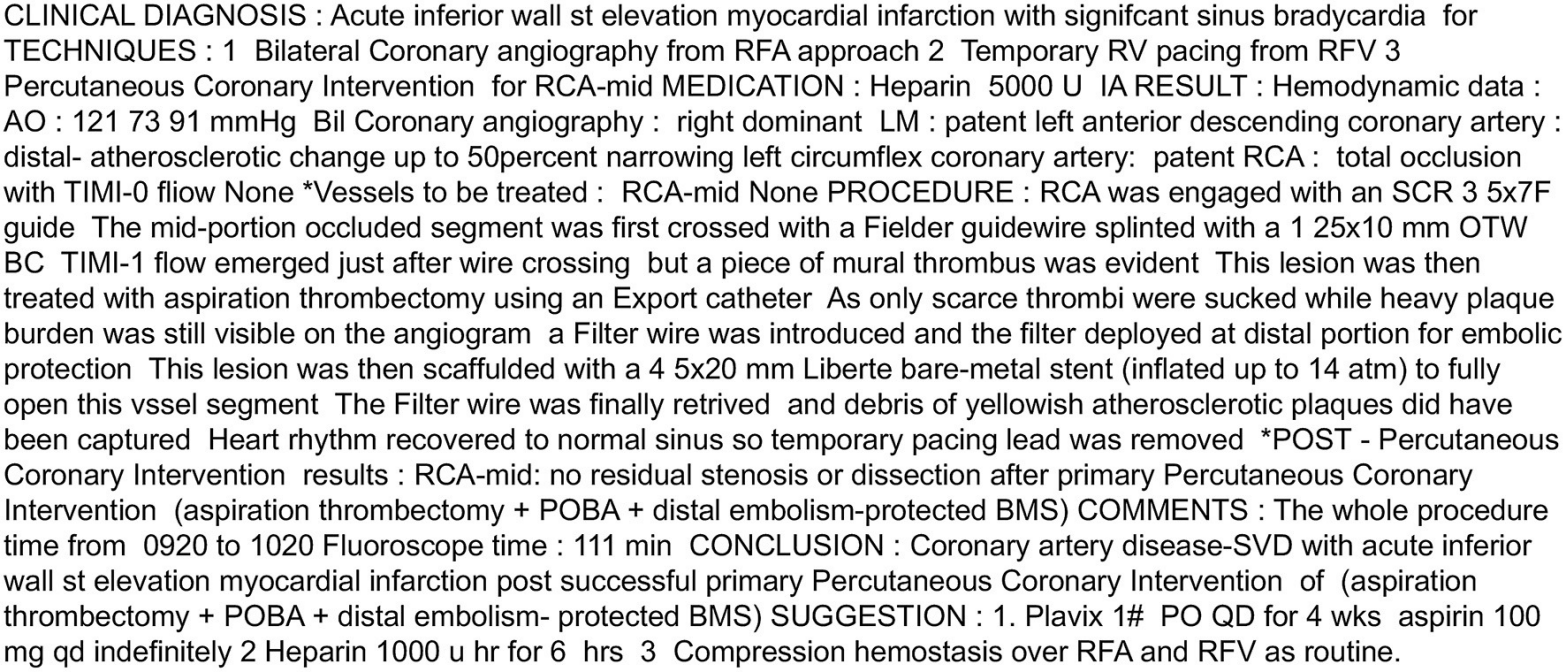
Example of the coronary catherization reports in our hospital.

**Figure 3:**
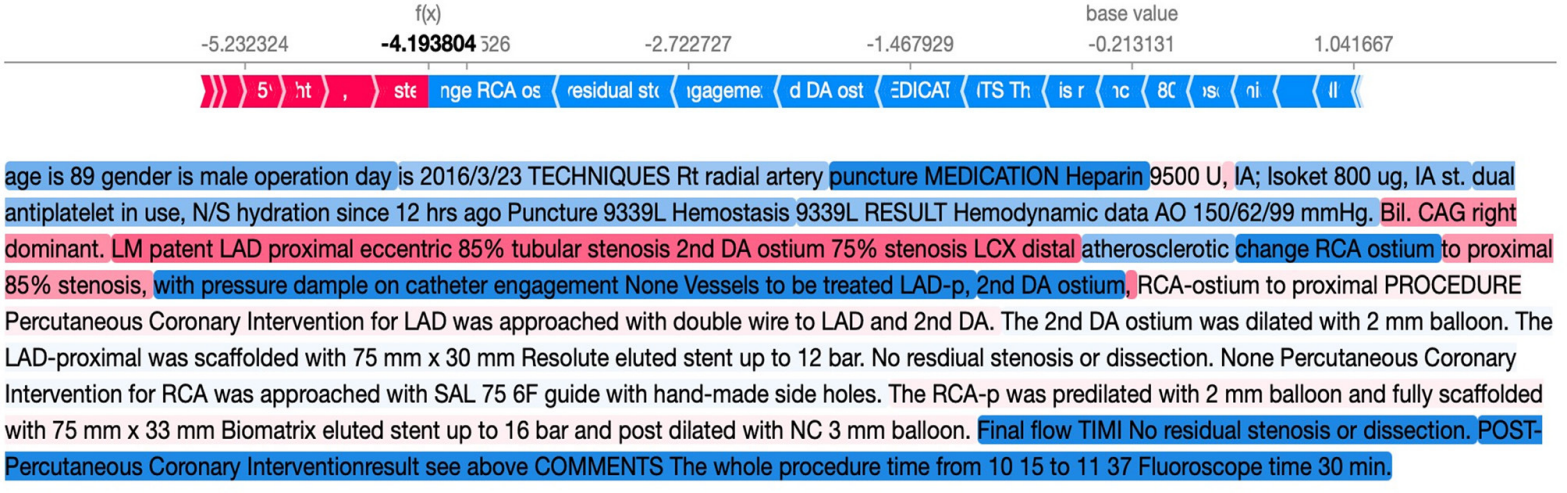
Example of the SHAP text plot for our reports. The words in red suggest that the word helps to predict mortality, whereas the words in blue suggest prediction of survival. SHAP, SHapley Additive explanation.

To provide interpretability for the BERT models, we applied SHapley Additive explanation (SHAP) to explain the model (12). SHAP assigned importance to a feature by approximating the effect of removing a variable from the original data. The model first passed the data with all features masked to generate a “base value,” and then subsequently mapped random coalition of texts and predicted the given segments. By comparing large amounts of prediction values from different coalitions, the SHAP values of each segment were generated. The difference between the SHAP values and traditional feature importance is that feature importance only indicates its global effectiveness toward the model, whereas SHAP values not only reflect the importance of each data point but also indicate whether the feature positively or negatively impacted the model. For clarity, an example of the original coronary catheterization reports and the SHAP text plot is shown in Figures 2, 3.

### Model Implementation

We deployed our model in the hospital's health information system, but the deployment was not open to the public due to data security reasons. However, the source code can be found at https://github.com/YowKuan/CAD_Prediction_API.

### Comparison With the RSS

To achieve a power of 0.9 and significant level of 0.05 for AUC comparison, at least 17 deaths and 130 lived patients were required. Therefore, we randomly selected 300 patients (41 deaths, 259 patients) from the dataset to calculate their RSS. We compared the AUC of our model for 5-year mortality and 5-year cardiovascular mortality to those of RSS. Net reclassification improvement (NRI) and integrated discrimination improvement (IDI) were used to evaluate the improvement in the predictive power of our final model compared with the RSS.

### Ethics Statement

This study was reviewed and approved by the Institutional Review Board of Taichung Veterans General Hospital. Written informed consent was not required for this study, in accordance with the local legislation and institutional requirements.

### Statistical Analysis

Continuous variables are reported as mean ± standard deviation, and categorical variables are reported as numbers with proportions. The Kruskal–Wallis test or chi-square test was performed for comparison, as appropriate. The probability produced by our model was divided into tertiles and the Cox proportional hazard model was used to assess the prognostic value. The AUC of each model was compared using DeLong's method. A *p*-value of < 0.05 was considered statistically significant. Analyses were performed using the R 3.4 software (The R Project for Statistical Computing, Vienna, Austria) and Python (version 3.6).

## Results

### Characteristics of Enrolled Patients

A total of 11,576 patients were included in the analysis. A total of 1,411 patients (12.2%) died within 5 years after undergoing coronary angiography, and 664 patients (5.8%) died of cardiovascular causes within 5 years after coronary angiography. The median follow-up time was 1,338 days (interquartile range, 659–2,302 days). Baseline characteristics of the cohort are presented in Table 1. In this cohort, the mean age was 65.5 years old and ~75% of the patients were male. Approximately 40% of the patients had a history of cardiovascular disease before undergoing coronary angiography and ~25% of the patients underwent coronary angiography because of ACS, and the remaining 75% with chronic coronary syndrome (CCS) underwent the procedure due to persistent angina despite medication use. According to the report of coronary angiography, ~29% of patients had non-obstructive CAD, and ~71% of the patients had obstructive CAD followed by PCI. The radial artery approach was used for coronary angiography in 67% of patients. The baseline characteristics were distributed equally, without significant differences between the training, validation, and test datasets.

**Table 1:** Baseline characteristics of the study population.

	**Training set**	**Validation set**	**Test set**	***p*-value**
Number	6,946	2,315	2,315	
Age (years)	65.5 ± 12.1	65.4 ± 11.2	65.6 ± 11.2	0.760
Male (*n*; %)	5,293 (76.2%)	1,806 (78.0%)	1,764 (76.2%)	0.184
CAD history (*n*; %)	2,737 (39.4%)	926 (40.0%)	923 (39.9%)	0.391
**Indication of coronary angiography**				
ACS (*n*; %)	1,703 (24.5%)	583 (25.2%)	548 (23.7%)	0.486
^*^Angina (*n*; %)	5,243 (75.5%)	1,732 (74.8%)	1,776 (76.3%)	
Number of coronary arteries with significant stenosis^**^( ***n*** **, %)**				
0	2,075 (29.9%)	675 (29.2%)	731 (31.6%)	0.310
1	2,165 (31.1%)	750 (32.4%)	708 (30.6%)	
2	1,819 (26.2%)	572 (24.7%)	609 (26.3%)	
3	887 (12.8%)	318 (13.7%)	267 (11.5%)	
Radial access^***^	4,722 (68.0%)	1,536 (66.3%)	1,528 (66.0%)	0.124
5-year CV mortality	407 (5.9%)	136 (5.9%)	119 (5.1%)	0.387
5-year all-cause mortality	847 (12.2%)	282 (12.2%)	282 (12.2%)	

* *Patients with chronic coronary syndrome, but persistent angina despite medication use*.

** *Significant stenosis defined as stenosis ≥50%*.

*** *Radial artery access for coronary catherization*.

*ACS, acute coronary syndrome; CABG, coronary artery bypass graft; CAD, coronary artery disease; CV, cardiovascular; PCI, percutaneous coronary intervention*.

### Model Performance

**Figure 4:**
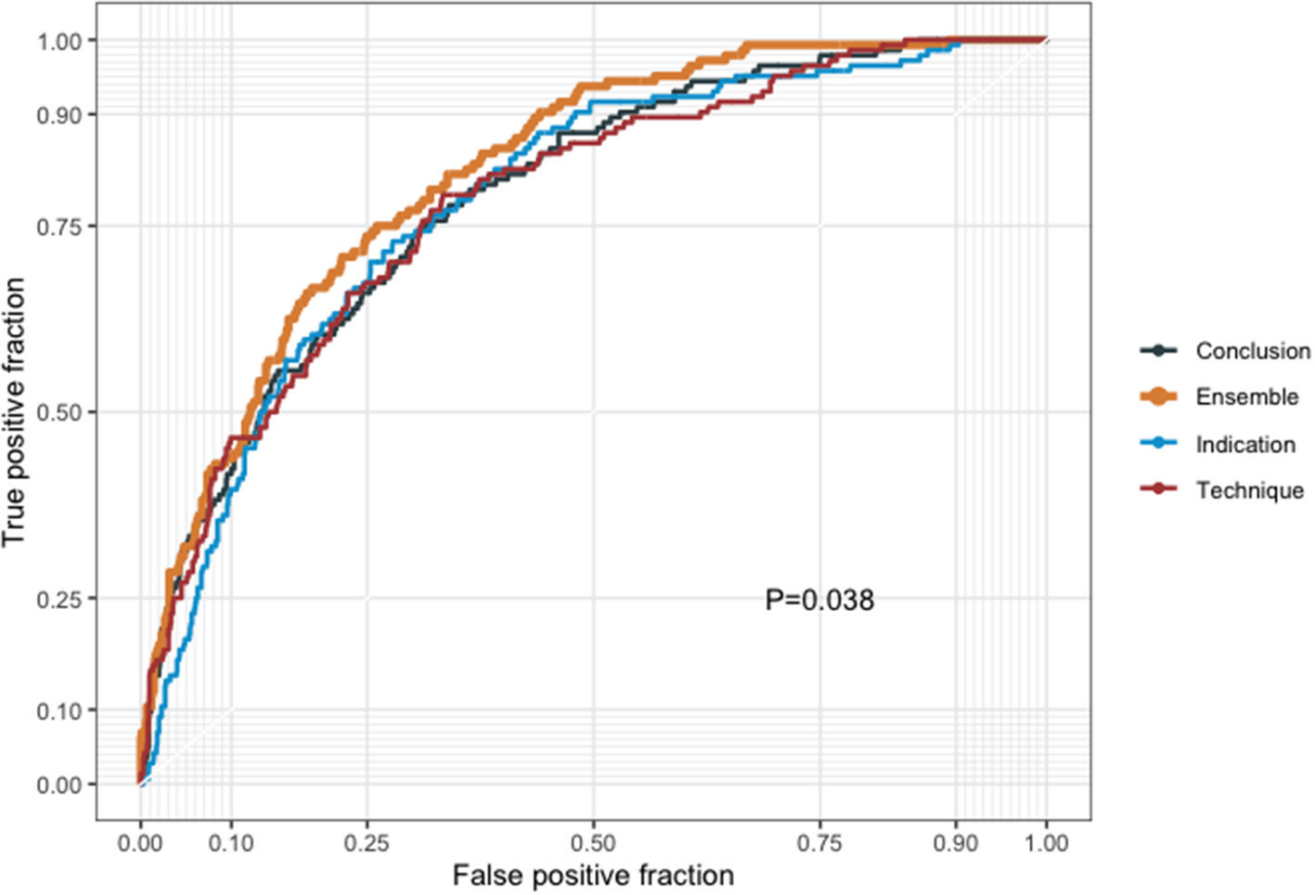
Receiver operating characteristic curves of the results from our model training with the indication, technique, and conclusion parts alongside the results from the combination of the three models.

For the 5-year all-cause mortality prediction, the model trained with the conclusion part had the highest positive predictive value, whereas the model trained with the technique part had the highest negative predictive value (Table 2). The AUC for the model trained with the indication part was 0.784 (95% CI, 0.747–0.822); the AUC for the model trained with the technique part was 0.784 (0.746–0.823); and the AUC for the model trained with the conclusion part was 0.791 (0.753–0.828). The AUC of the ensemble model was 0.822 (0.790–0.855), which was significantly higher than that of the separated models (*P* < 0.001), as shown in Figure 4.

**Table 2:** Model performance.

**Model**	**PPV (95% CI)**	**NPV (95% CI)**	**AUC (95% CI)**
Indication	0.782 (0.756–0.807)	0.631 (0.547–0.710)	0.784 (0.747–0.822)
Technique	0.597 (0.567–0.627)	0.826 (0.754–0.884)	0.784 (0.746–0.823)
Conclusion	0.804 (0.779–0.828)	0.604 (0.519–0.684)	0.791 (0.753–0.828)
Ensemble model	0.782 (0.756–0.807)	0.687 (0.605–0.762)	0.822 (0.790–0.855)

*AUC, area under the receiver operating characteristic curve; NPV, negative predictive value; PPV, positive predictive value*.

**Figure 5:**
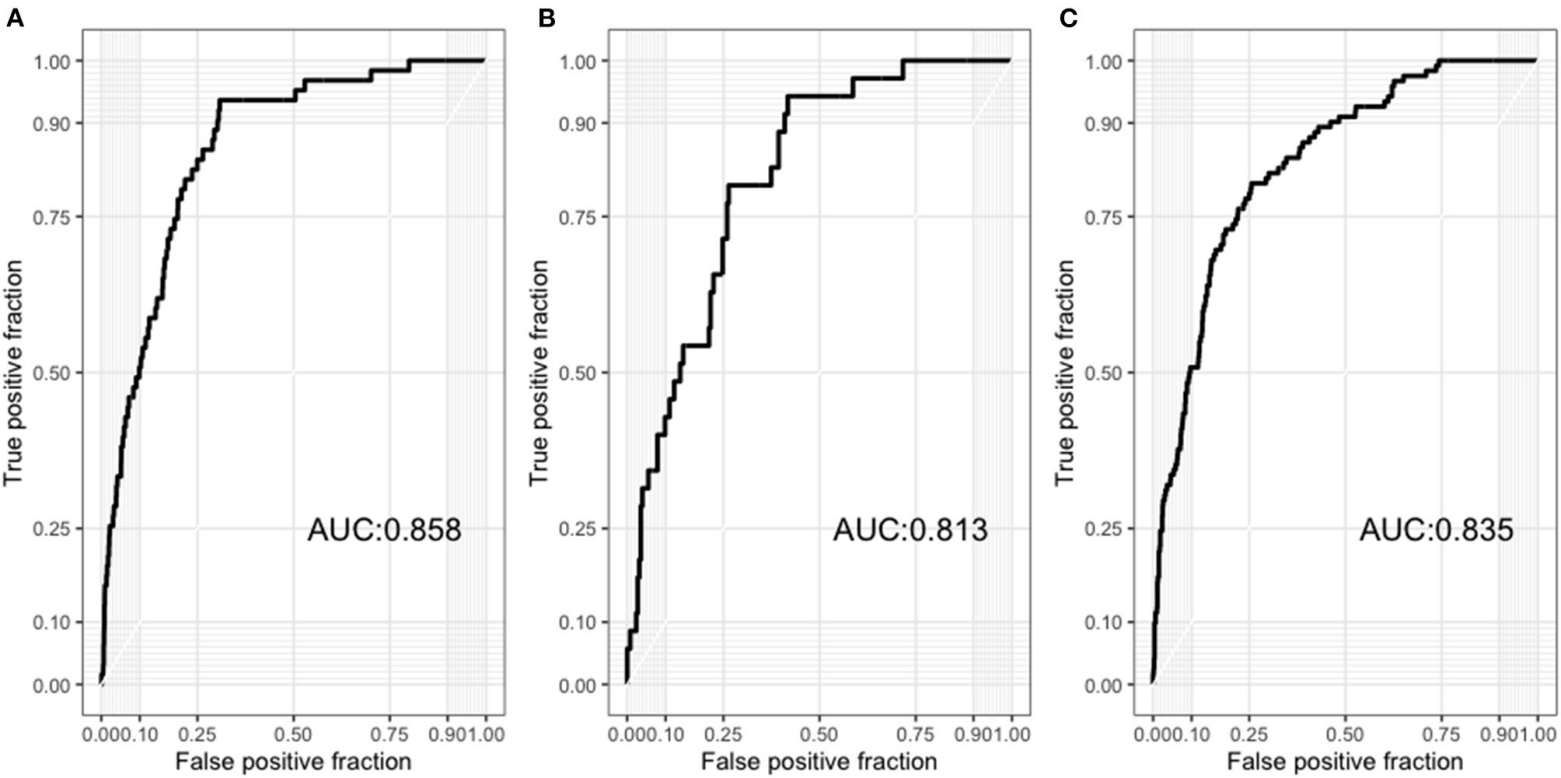
**(A)** Receiver operating characteristic curves of the results from our model to predict 5-year all-cause mortality among patients with acute coronary syndrome. **(B)** Receiver operating characteristic curves of the results from our model to predict 5-year all-cause mortality among patients with chronic coronary syndrome. **(C)** Receiver operating characteristic curves of the results from our model to predict 5-year cardiovascular mortality in all patients.

Therefore, we used the best model (ensemble model) for further analysis. To determine whether the presence of ACS affects model performance, we divided our dataset into patients with ACS and those with CCS. The AUC for patients with ACS and CCS was 0.835 (95% CI, 0.799–0.872) and 0.813 (0.748–0.879), respectively (Figures 5A,B). Regarding the 5-year cardiovascular mortality prediction, the AUC of our best model was 0.858 (95% CI, 0.816–0.900) (Figure 5C).

### Prognostic Value of the Model

**Figure 6:**
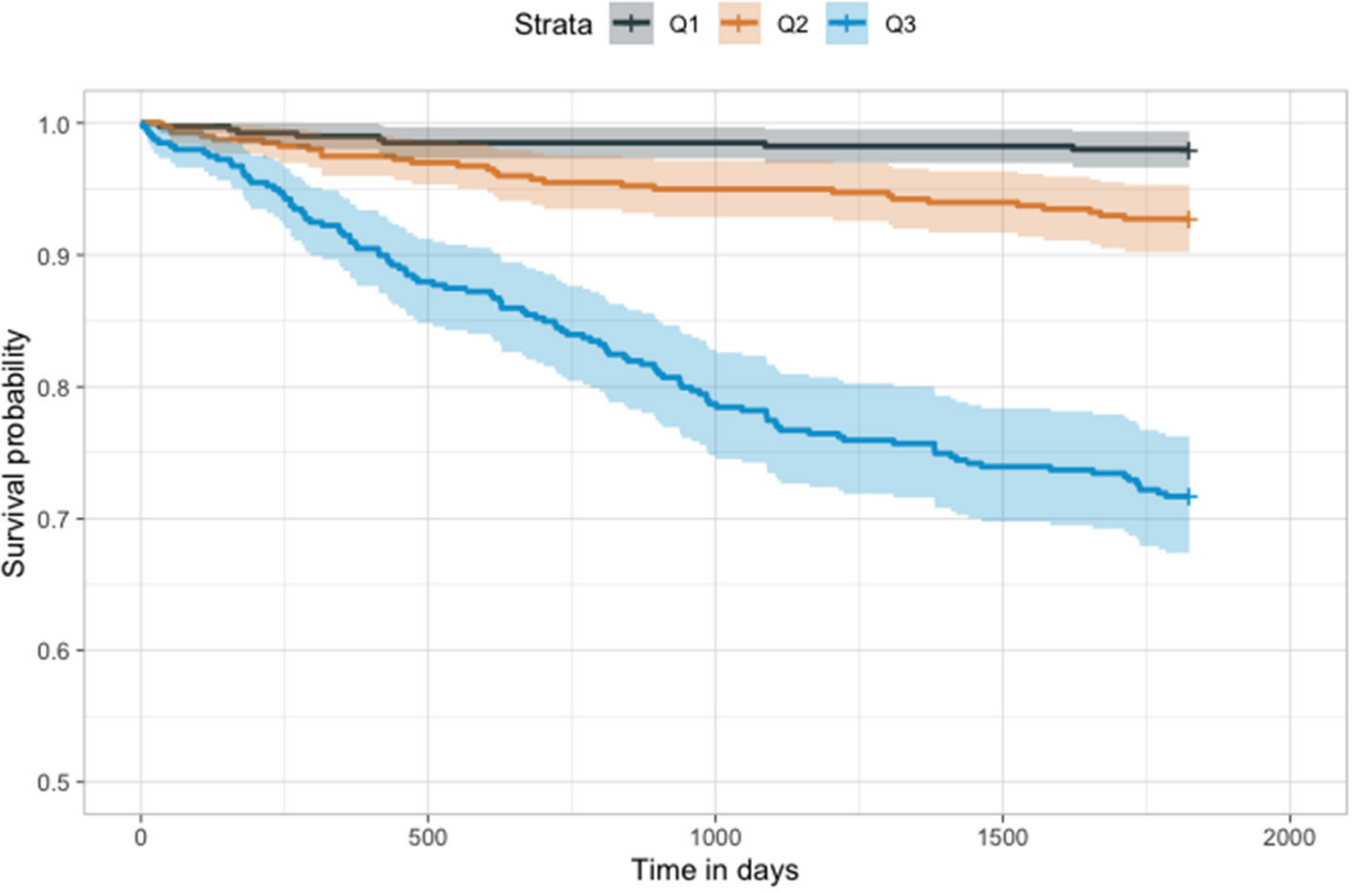
Kaplan–Meier plot of our model. The results of our model were divided into tertiles. Higher tertiles had significantly lower survival probability (*p* < 0.001).

In the Cox model, we found that the probability produced by our model was a significant predictor of mortality (*P* < 0.001). Patients in both the highest tertile (Hazard ratio: 16.3; 95% CI, 7.9–33.5) and those in the second highest tertile (Hazard ratio, 3.7; 95% CI, 1.7–8.5) had a higher mortality risk than patients in the lowest tertile (Figure 6).

### Comparison With the RSS

**Figure 7:**
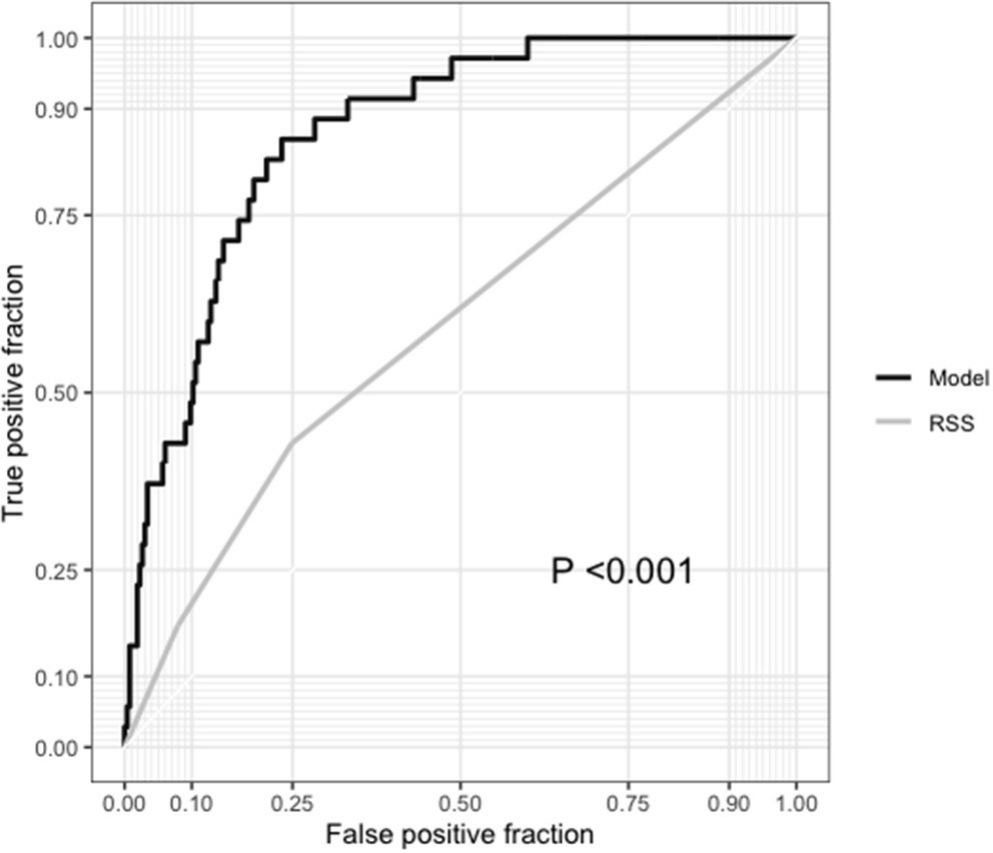
Comparison between the receiver operating characteristic curves of our model and the residual SYNTAX score (RSS).

Among the 300 patients with complete RSS, our model showed a significantly higher AUC than the RSS (0.867 vs. 0.590, respectively; *P* < 0.001) for 5-year all-cause mortality, and the IDI (0.272; 95% CI, 0.172–0.373; *P* < 0.001), and NRI (0.213; 95% CI, 0.005–0.421; *P* = 0.04) indices also showed improvement in predictive ability compared with the RSS (Table 3). For cardiovascular mortality our model also had a significantly higher AUC than the RSS (0.880 vs. 0.649, respectively; *P* < 0.001) and showed better predictive ability than the RSS (IDI, 0.229; 95% CI, 0.127–0.373; *P* < 0.001; NRI, 0.337; 95% CI, 0.005–0.421; *P* = 0.001; Table 4 and Figure 7).

**Table 3:** Comparison of performance for all-cause mortality prediction between the RSS and Ensemble model.

**Model**	**AUC (95% CI)**	***p*-value**	**IDI (95% CI)**	***p*-value**	**NRI (95% CI)**	***p*-value**
RSS	0.590 (0.503–0.684)					
Ensemble model	0.867 (0.813–0.921)	<0.001	0.272 (0.172–0.373)	<0.001	0.213 (0.005–0.421)	0.04

*AUC, area under the receiver operating characteristic curve; CI, confidence interval; IDI, integrated discrimination improvement; NRI, net reclassification improvement; RSS, residual SYNergy between PCI with TAXUS and Cardiac Surgery score*.

**Table 4:** Comparison of performance for cardiovascular mortality prediction between the RSS and the model.

**Model**	**AUC (95% CI)**	***p*-value**	**IDI (95% CI)**	***p*-value**	**NRI (95% CI)**	***p*-value**
RSS	0.649 (0.535–0.764)					
Ensemble model	0.880 (0.873–0.925)	<0.001	0.229 (0.127–0.332)	<0.001	0.337 (0.131–0.543)	0.001

*AUC, area under the receiver operating characteristic curve; CI, confidence interval; IDI, integrated discrimination improvement; NRI, net reclassification improvement; RSS, residual SYNergy between PCI with TAXUS and Cardiac Surgery score*.

## Discussion

In the current study, we constructed a predictive tool for patients undergoing coronary angiography to predict the 5-year all-cause mortality and 5-year cardiovascular mortality. To the best of our knowledge, this is the first study to utilize a deep learning NLP algorithm to build a predictive model for patients undergoing coronary angiography. Since interventional cardiologists routinely write coronary catheterization reports after coronary angiography, models using these reports as inputs do not require parameters to calculate the score, which is considered more clinically convenient.

Prior predictive models have some limitations in clinical applications (13). For patients who present with ACS, the GRACE score (14), which is composed of age, creatinine level, heart rate, systolic blood pressure, Killip class, abnormal cardiac enzyme level, and ST elevation shown on EKG, is frequently used to estimate in-hospital mortality. The risk scores showed good discriminative ability with a C-statistic of 0.75 in the original article; however, if one of these parameters was not documented in the EHR, then the score could not be used. For patients with complex CAD, the SYNTAX score (4), which is calculated based on the coronary artery lesions to quantify the atherosclerosis burden, is well-validated and widely used to predict adverse outcomes after PCI; however, the calculation requires experts to review coronary angiography records and reports, which need considerable time and effort. The advantage of using the text content of coronary catheterization reports to construct models is that they do not require predefined parameters. In coronary catheterization reports, interventional cardiologists describe in detail the indications for the procedure, technique, and conclusion, which are all important information for outcomes. Our model demonstrated that using text content from reports can achieve good performance and even outperformed the RSS score for mortality prediction.

Since there are heterogenicity in our datasets, comprised of both patients with ACS and patients with CCS. We separately investigated our model for these two subsets of patients and our model still showed good performance in both patients with ACS and CCS. Currently, predive models that proved to be effective both in patients with ACS and CCS are rare, which give our model advantage for future use in clinical practice. However, an external validation with a larger sample size is warranted in the future.

The novelty of our model is that we used the state-of-the-art BERT model to construct our predictive tool. Numerous machine learning algorithms have been developed in the cardiovascular field (15). The majority of them use discrete data such as values from lab report and patient demographic data as inputs to construct the models (16). Recently, deep learning models using medical images such as cardiac MRI and cardiac CT scans have been proposed to predict cardiovascular prognosis (17). However, deep learning models using the NLP technique are scarce and mostly applied to radiological reports. Zheng et al. developed an NLP algorithm to identify pulmonary nodules and the associated characteristics with high accuracy (18). Furthermore, a recent study compared different machine learning NLP methods to classify radiology reports in orthopedic trauma for injuries and found that BERT NLP outperformed traditional machine learning models and rule-based classifiers for Dutch radiology reports in orthopedic trauma (19). However, no NLP-based deep learning algorithm has been reported in the field of cardiovascular disease research. As text reports comprise a large proportion of EHRs and have abundant valuable information embedded as unstructured data, we believe that a model utilizing the deep learning NLP algorithm is valuable and can have a substantial clinical impact in the future.

In the BERT-base model, the limitation on the maximum input length was 512 (20). Since the reports we used had hundreds to thousands of words, we divided the reports accordingly into three parts, namely, indication, technique, and conclusion. Interestingly, we found the AUCs to be similar between the three models trained with indication, technique, and conclusion part, separately. However, there were still difference in the performance of these three models. The model trained with the technique content had the highest negative predictive value, whereas the model trained with the conclusive content had the highest positive predictive value, and together the performance improved when we ensembled all three models. This may imply that each part of the report has a unique role in the final prediction. BERT-based models have been criticized for having limitations with a maximum input length of 512. Since clinical text reports often have thousands of words, the application of BERT-based models in the medical domain is limited. Therefore, our approach to divide the text reports into meaningful parts and then ensemble models trained with different parts of the texts can serve as an alternative solution to address this limitation.

There are several limitations to the current study. First, since our cohort was retrospective and single center in nature, an external validation study with an independent dataset from another hospital is needed to prove the generalizability of the model. Second, the coronary catheterization reports need to be divided into three parts before fitted into the model. However, since coronary catheterization reports are required to describe the indication of the procedure, the vascular lesions, the techniques used, and a summary, clinicians could divide their reports into these three parts according to their report format. No standardized method of reporting or specific format was required to fit our model if the reports contained descriptions of indications, techniques, and conclusions. Lastly, since our model was trained with English text reports, our model can only be applied to English coronary catheterization reports.

In conclusion, we developed a predictive model using cardiac catheterization reports as inputs to predict the mortality in patients undergoing coronary angiography. The model showed excellent performance in predicting the 5-year mortality of patients undergoing coronary angiography. For future research, we will add more clinical information to our model to investigate its influence on model performance. In addition, we will collect more relevant cardiovascular outcomes, including re-admission for PCI and recurrent stenosis, to fine-tune our model for more relevant cardiovascular outcomes prediction.

## Data Availability Statement

The raw data supporting the conclusions of this article will be made available by the authors, without undue reservation.

## Ethics Statement

The studies involving human participants were reviewed and approved by Institutional Review Board of Taichung Veterans General Hospital. Written informed consent for participation was not required for this study in accordance with the national legislation and the institutional requirements.

## Author Contributions

Y-HL and F-PL: full access to all the data in the study and take responsibility for the integrity of the data and the accuracy of the data analysis. Y-HL: concept and design. Y-HL, I-TL, and Y-KL: drafting of the manuscript. Y-WC: critical revision of the manuscript for important intellectual content. Y-KL and Y-HL: statistical analysis. All authors acquisition, analysis, or interpretation of data.

## Funding

This work was supported by Taichung Veterans General Hospital, Taichung, Taiwan (Grant number TCVGH-1103501B), the National Health Research Institute (Grant number NHRI-EX110-10927HT), and the Ministry of Science and Technology (Grant number MOST 109-2634-F-002-032).

## Conflict of Interest

The authors declare that the research was conducted in the absence of any commercial or financial relationships that could be construed as a potential conflict of interest.

## Publisher's Note

All claims expressed in this article are solely those of the authors and do not necessarily represent those of their affiliated organizations, or those of the publisher, the editors and the reviewers. Any product that may be evaluated in this article, or claim that may be made by its manufacturer, is not guaranteed or endorsed by the publisher.
